# Initial psychological impact of COVID-19 and its correlates in Indian Community: An online (FEEL-COVID) survey

**DOI:** 10.1371/journal.pone.0233874

**Published:** 2020-05-29

**Authors:** Mohit Varshney, Jithin Thomas Parel, Neeraj Raizada, Shiv Kumar Sarin

**Affiliations:** 1 Department of Psychiatry, Institute of Liver and Biliary Science (ILBS), New Delhi, India; 2 College of Nursing, Institute of Liver and Biliary Science (ILBS), New Delhi, India; 3 Department of Epidemiology and Clinical Research, Institute of Liver and Biliary Science (ILBS), New Delhi, India; 4 Department of Hepatology, Institute of Liver and Biliary Science (ILBS), New Delhi, India; Faculty of Science, Ain Shams University (ASU), EGYPT

## Abstract

**Background:**

The pandemic of Corona Virus (COVID-19) hit India recently; and the associated uncertainty is increasingly testing psychological resilience of the masses. When the global focus has mostly been on testing, finding a cure and preventing transmission; people are going through a myriad of psychological problems in adjusting to the current lifestyles and fear of the disease. Since there is a severe dearth of researches on this issue, we decided to conduct an online survey to evaluate its psychological impact.

**Methods:**

From 26^th^ to 29^th^ March an online survey (FEEL-COVID) was conducted using principles of snowballing, and by invitation through text messages to participate. The survey collected data on socio-demographic and clinical variables related to COVID-19 (based on the current knowledge); along with measuring psychological impact with the help of Impact of Event–revised (IES-R) scale.

**Results:**

There were a total of 1106 responses from around 64 cities in the country. Out of these 453 responses had at least one item missing; and were excluded from the analysis. The mean age of the respondents was around 41 years with a male female ratio of 3:1 and around 22% respondents were health care professionals. Overall approximately one third of respondents had significant psychological impact (IES-R score > 24). Higher psychological impact was predicted with younger age, female gender and comorbid physical illness. Presence of physical symptoms and contact history predicted higher psychological impact, but did not reach statistical significance.

**Conclusion:**

During the initial stages of COVID-19 in India, almost one-third respondents had a significant psychological impact. This indicates a need for more systematic and longitudinal assessment of psychological needs of the population, which can help the government in formulating holistic interventions for affected individuals.

## 1. Introduction

Corona is a single stranded RNA virus that had its roots into the world from almost 60 years since its discovery in late 1960s. Corona viruses belong to the Corona viridae family in the Nidovirales order. The nomenclature of the Corona virus is named after the crown-like spikes on the outer surface of the virus structure. [1] The virus has been infecting animals like chickens and pigs but there was no major human contraction to humans. [2] Earlier, the allied viruses of the same family like the Severe acute respiratory syndrome coronavirus SARS-CoV in 2003, Human corona virus HCoV NL63 in 2004 [3], HKU1 in 2005 [4], Middle east respiratory (MERS) in 2012, have shown their outbreaks and now the novel version of this virus has presented a threat of unmatched severity. According to the classification of International Taxonomy of Viruses (ICTV) has referred this novel pathogen as SARS-CoV-2 (formerly known as 2019-nCoV) in 2019. [5,6] The first case was identified in the city of Wuhan, a Chinese seafood market and since then it has been exponentially increasing with an evident human to human contact via respiratory droplets while sneezing and coughing. [7] The mode and transmission and other related details about the virus continue to be updated in every few weeks, leading to enhanced uncertainty. [8] During this period most of the research has been focused on understanding and preventing transmission; exploring treatment options and issues with global governance. However we think that the psychological impact of this pandemic like stress and anxiety among the general population is also a grave concern. [9] A study from China suggesting that more than half of the participants had a significant psychological impact of the COVID-19 pandemic. Another recent study from Denmark reported psychological well-being as negatively affected. In the United States nearly half were found to be anxious as per the survey conducted by the American Psychiatric Association. [10–13] The same has not been studied in Indian population systematically; except anecdotal discussions and case reports. [14]

In Indian subcontinent, as of 30 March 2020, according to the Ministry of Health & Family Welfare (MoHFW), a total of 1071 COVID-19 positive cases (including 49 foreign nationals) were reported in 27 states/union territories. These include 99 cases that were cured / discharged, one person who has migrated and 29 deaths. [2] Hospital isolation of all confirmed cases, tracing and home quarantine of the contacts is on-going. In India, spread of the initial disease could be traced mainly to the foreign nationals who visited the country as tourists from the disease affected countries and secondly due to the mass immigration of Indian nationals from abroad; due to the fear of infection. As the pandemic outbreak in India was on-going, the Government of India took stringent measures to limit the cases by far in that stage only, by initiating a major lockdown pan-India and also by shifting the immigrants to the special quarantine facilities prepared by the Indian Military directly from the airports and seaports for a minimum of 14 days. Community health teams were also launched to spread awareness about the chances of spread and precautionary measures that one can use to protect themselves and others. [15]

During the early stages of the pandemic in India, this study was focused mainly to assess its psychological impact. The lives of people were drastically affected with lock-down and fear related to the disease’s potential effects and transmission [15]. The fear due to the contraction of COVID -19 is on the rise because of the death tolls and global spread. [16,17]. Hence, this study attempted to find the initial psychological impact of COVID-19 among general public; and understand its relationship with physical symptoms. This can potentially help policy makers in formulating comprehensive interventions.

## 2. Methodology

The study has been approved by the Institutional Ethics Committee at Institute of Liver and Biliary Sciences, New Delhi (letter no: IEC/2020/73/MA04). A cross sectional survey design was decided to assess the initial psychological impact of COVID-19, (fears worries and impairment in sleep). We collected data using an online (anonymous) survey platform (Survey Monkey) as per Indian Government’s recommendations to minimise face-to-face or physical interaction as citizens continue to isolate themselves at home. Potential respondents were invited through a text message, which lead them to a survey monkey page (designed by IT team at ILBS, New Delhi). All people who have registered at ILBS (2009 to present) since the inception were sent the SMS for participation in the FEEL-COVID survey.

Additionally, using the principles of snowballing, the link was circulated by the investigators through social media for capturing data from English speaking general population (who have some access to Internet). An effort was made to capture healthcare workers who have handled patients / potential patients. Additionally, family member of patients suffering from Liver disease, being screened in Institute of Liver and Biliary Sciences, were requested to take the survey while waiting for their consultation. During offline requests all standard social distancing protocols were maintained as directed by Indian government. We collected data anonymously, without collecting information that could identify the respondents. The period of data collection was between 26^th^ and 29^th^ March 2020.

### 2.1 Study questionnaire

Once the user clicked on the link they were given information about the nature and purpose of survey on the first page. Subsequently, if they consented to participate, they were taken to the next page (first section) of the survey. The first part of the study questionnaire collected socio-demographic information (age, gender, occupational status, city of residence, type of family) and information regarding physical symptoms like presence of cough, cold, head ache breathing difficulty, fever and fatigue related to Coronavirus disease. Contact history variables included close contact with an individual with confirmed COVID-19, indirect contact with an individual with confirmed COVID-19, and contact with an individual with suspected COVID-19 or infected material; and any foreign travel in the last 14 days. Participants were also asked about being a healthcare worker and if they had a known pre-existing medical or psychiatric illness.

The second part of the survey was adopted from Impact of Event scale–revised (IES-R). This tool comprised of 22-items questionnaire which measure the effect of routine life stress, everyday traumas and acute stress. For all questions, scores could range from 0 through 4. Categorization of the score ranges from 24 to 32, 33 to 36 and more than 37 which signify mild, moderate and severe psychological impact respectively. [18,19]. Among this scale, the Intrusion subscale is mean item response of items 1, 2, 3, 6, 9, 14, 16, 20. The Avoidance subscale is the mean item response of items 5, 7, 8, 11, 12, 13, 17, 22. The Hyperarousal subscale is the mean item response of items 4, 10, 15, 18, 19, 21.

Separately, the data on actual number of confirmed cases of COVID-19 and deaths in the Country was accessed through Government of India website for general public which was available in the website URL address “https://www.mygov.in/covid-19”. For the purpose of this study we accessed the above website till 31^st^ March 2020.

### 2.2 Statistical analysis

Descriptive statistics were conducted for the socio-demographic variable and clinical parameters (like physical symptoms and contact history). Normality of data was assessed using Shapiro-Wilk test. The scores of the IES-R and subscales were expressed as mean and standard deviation. We used linear regression to calculate the univariate associations between socio-demographic characteristics, physical symptom contact history variables, additional health information variables and IES-R score. All tests were two-tailed, with a significance level of p < 0.05. Statistical analysis was performed using SPSS Statistic 22.0 (IBM SPSS Statistics, New York, United States).

## 3 Results

### 3.1 COVID-19 pandemic from 1^st^ February 2020 to 30^th^ March 2020

**Fig 1:**
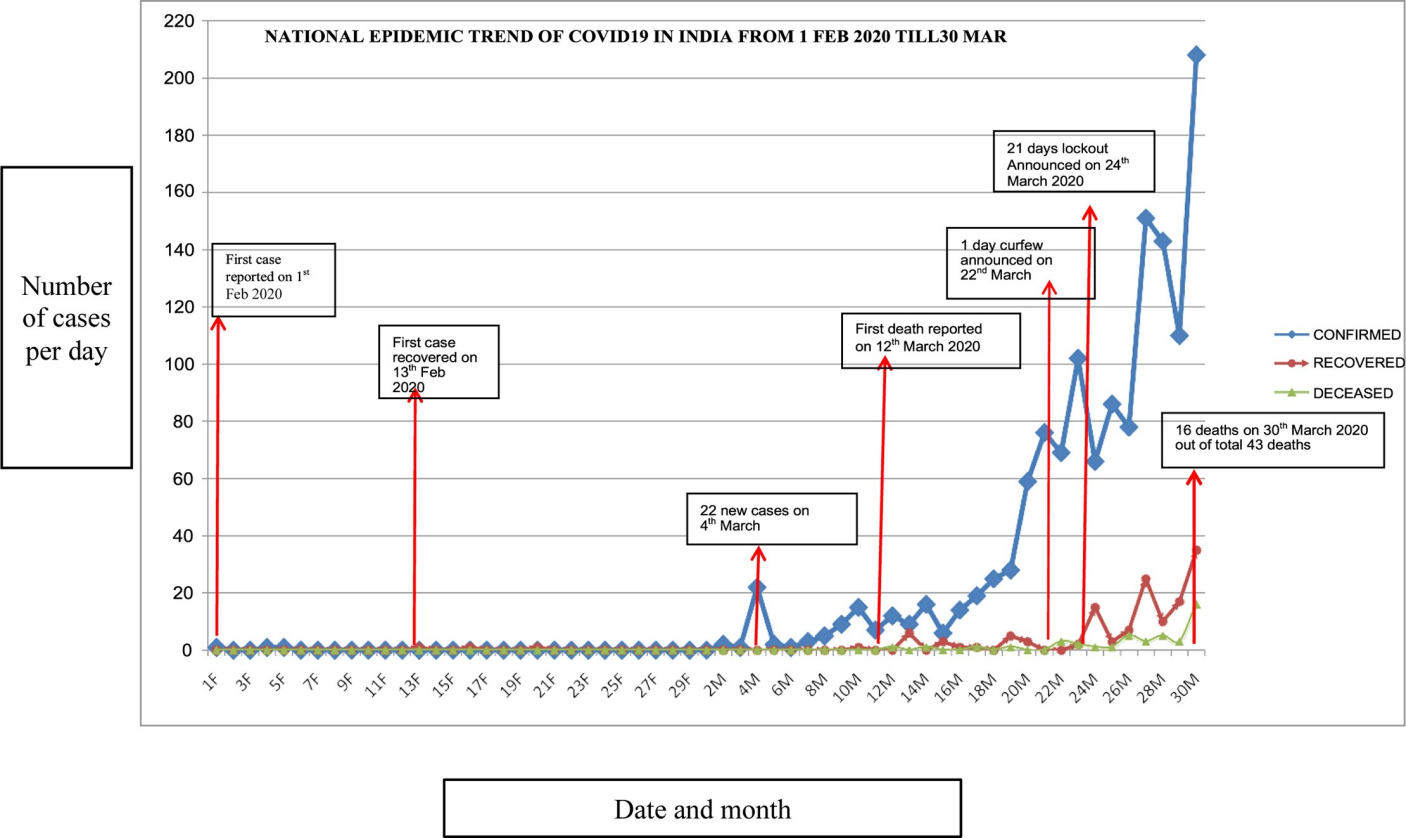
Timeline of events during the early phase of pandemic in India. *(Source*: https://www.mygov.in/covid-19).

During the very early period, Fig 1 depicts the progression of number of cases of COVID-19 from 1^st^ February 2020 to 30^th^ March 2020 in India. The figure also has a timeline of events (first case, first recovered case, first death and curfew announced) to get a perspective of results during the initial period of COVID-19 in India. The first case of COVID-19 was reported on 1st February 2020 in India. Thereafter there was a significant increase in the number of the confirmed, recovered and deceased individuals due to coronavirus outbreak up to 30th march 2020. (“https://www.mygov.in/covid-19). At the time of conducting the survey, the number of cases was building up.

### 3.2 Characteristics of survey respondents

A total of 1106 responses were obtained in the study duration through the survey monkey platform. Out of these 453 had at least one item missing in the psychological impact related responses and were excluded from analysis. The final analysis was done on rest of the 653 respondents. The mean age of the respondents were 41.82 years (SD = 13.85; range = 18–82) with a male preponderance [491(75.2%)]; among which 145 participants (22.2%) were health professionals. Most of the respondents 400(61.3%) belong to nuclear families and 257(39.3%) respondents had reported a history of physical illness; including 125 (19.1%) with a history of known Liver disease.

### 3.3 Psychological impact and subscales

The psychological impact of COVID-19 outbreak, as measured by IES-R scale, revealed a mean score of Mean of 19.79 ((SD) = 13.89) and Median of 18.00. As it can be seen from the Table 1, most of the respondents 436 (66.8%) had minimal psychological impact 436 (66.8%) in reaction to COVID-19 outbreak. Around 98 (15.0%) had mild psychological impact (IES-R score of 24–32) and 36 (5.5%) had moderate psychological impact (IES-R score of 33–36) However, 83 (12.7%) reported severe psychological impact (IES-R score of >36). (Table 1)

**Table 1:** Frequency and percentage distribution of psychological impact in response to COVID-19 outbreak.

S.no	Scale derived values (N = 653)	Categories	Frequency	Percentage
1	Impact of event scale	Minimal (0–23)	436	66.8
		Mild (24–32)	98	15.0
		Moderate (33–36)	36	5.5
		Severe (>36)	83	12.7
Subscales (Range of score)		Items	Mean (SD)	Median
2	Intrusivity subscale (0–32)	Q 1,2,3,6,9,14,16,20	6.82 (5.68)	6.00
3	Avoidance subscale (0–32)	Q 5,7,8,11,12,13,17,22	7.94 (5.81)	8.00
4	Hyper-arousal subscale (0–24)	Q 4,10,15,18,19,21	5.01 (4.16)	4.00

(SD = Standard deviation; Q = Question number from IES-R scale)

### 3.4 Correlation of psychological impact with clinical variables

#### 3.4.1 Association of demographic variables and impact on psychological health (Table 2)

Linear regression showed that there was a statistically significant association found between male counter parts and minimal psychological impact which ranges from 0 to 23 on IES-R Scale; and between age and psychological impact with higher age associated with lesser psychological impact. Moreover, there was a significant association between history of any physical illness and psychological impact. However, there were no statistically significant association between any other demographic or clinical variables.

**Table 2:** Association of demographic and clinical variables with psychological impact in response to COVID-19 outbreak (Univariate Linear regression).

S.no	Variables	N (%)	R^2^	Standardized Beta(95%CI)	P-Value
		Total IES–R score			
1	Age	Mean (653)-41.82yrs	.016	-.127 (-.211 - -.042)	.004*
2	Gender	Male– 491 (75.2)	.018	-.134 (-6.887–1.882)	.001*
		Female—154 (23.6)			
		Others– 8 (1.2)			
4	Health Professional	Yes—145 (22.2)	-.002	.001(-2.540–2.618)	.976
		No—508 (77.8)			
5	Type of family	Nuclear—400 (61.3)	.002	.042(-.991–3.411)	.281
		Joint—253 (38.7)			
6	Presence of any physical disease	Yes-257 (39.3)	.005	.071 (-.134–3.352)	.007*
		No-396 (60.7)			
7	Presence of known liver disease	Yes—125 (19.1)	.005	.072 (-.170–5.244)	.066
		No– 528 (80.9)			
Physical Symptoms					
1	Presence of cough	Yes-62(9.6)	.002	-.047(-3.913-.963)	.235
		No-591(90.4)			
2	Presence of Cold	Yes-34 (5.2)	.001	-.009(-3.552–2.828)	.824
		No-619 (94.8)			
3.	Presence of sore throat	Yes-51(7.9)	.002	.004(-2.576–2.830)	.926
		No- 602 (93.1)			
4.	Myalgia/body ache	Yes-48(7.4)	.001	-.014(-3.538–2.433)	.717
		No-605(93.6)			
5.	Presence of headache	Yes-87(7.9)	.005	-.073(-5.860-.174	.065
		No-566(92.1)			
6.	Fever (Temp>100F or 37.5C	Yes-17(1.6)	.001	.010(-6.143–7.982)	.798
		No-636(98.4)			
7.	Breathing difficulty	Yes-11(1.7)	.001	-.009(-5.598–4.393)	.813
		No-642(98.3)			
8.	Fatigue	Yes-52(8.0)	.004	-0.060(-4.873-.60.8)	.127
		No-601(92.0)			
Contact History in last 14 days					
1	International travel	Yes-9(1.4)	.003	-.055(-15.703–2.595)	.160
		No-644(98.6			
2	COVID– 19 infected area travel	Yes-20(3.1)	.002	.994(-4.194–24.194)	.994
		No-633 (96.9)			
3	Direct Contact with COVID-19 Positive	Yes-6(0.9)	-.001	-.014(-5.467–3.746)	.714
		No-644 (99.1)			

(SD = Standard deviation * = statistically significant; CI = Confidence Interval)

### 3.5 Physical symptoms and its association with impact on psychological health (Table 2)

As far as physical symptoms were concerned 62 (9.6%) respondents had reported the presence of cough. 34(5.2%) respondents reported presence of cold. However, diarrhoea was the least reported physical symptoms which accounts for merely in two (0.3%) respondents whereas headache was among 87 (12.3%) which was more frequently reported compared to other physical symptoms. Interestingly, sore throat and myalgia were present in 51(7.7%) and 48(7.4%) respectively. Only a few respondents had the symptoms of fever 17(2.6%) and breathing difficulty 11 (1.6%).

Univariate Linear regression revealed that there was a statistically significant association with presence of diarrhoea and the impact on their psychological health (p = 0.006). There was no statistically significant association between the physical symptoms such as cough, cold, headache, sore throat, myalgia, fever and breathing difficulty.

### 3.6 Contact history and its association with impact on psychological health

Only nine (1.4%) respondents had travelled during past fortnight, 20 (3.1%) had visited COVID-19 infected areas. 6(0.9%) had direct contact with the COVID-positive persons. There was no statistically significant association between contact history of the respondents and their impact on psychological health.

## 4 Discussion

The current study investigated the initial psychological impact of COVID-19 outbreak in Indian population. As the disease progressed, concerns regarding health, economy, and livelihood increased day-to-day. The findings of the pandemic’s impact on mental health could help inform health officials and the public to provide mental health interventions to those who are in need. This can guide researchers to plan prospective longitudinal studies for assessing treatment need. [20] There are mental health concerns like anxiety, worries and insomnia especially after the declaration of lockdown in India on 24^th^ March, 2020. Government of India has launched helpline numbers to provide guidance and counselling, in collaboration with different Institutes of national importance. [21] World Health Organization has urged to take the necessary precautions to tackle the negative impact of the spread of Coronavirus on psychological health and well-being. [22]

Overall, among the 653 respondents 33.2% had significant (mild / moderate /severe) psychological impact regarding COVID-19. This finding was different from the study conducted in china by Wang et al which reported 53.8% of respondents suffered a psychological impact from the outbreak, ranging from moderate to severe among 1210 respondents. [10] Since these findings were during the early phase of COVID-19 outbreak in the country, chances are they could have changed over time and hence, should be interpreted accordingly. In the past, during outbreaks such as ‘Ebola Virus’, individual and community at national and international had a major and wide spectrum of psychosocial impacts due to the sudden outbreak of the disease. It is likely that people are relating contracting the virus with a fear of falling sick, helplessness, hopelessness, stigma and even death. [23]

Providing psychological first-aid & counselling are quintessential during an epidemic. It helps in reducing the psychological distress and promoting adaptive coping strategies to deal with the situation. [24] Despite the efforts of WHO and other public health authorities to contain the COVID-19 outbreak, this time of crisis is generating stress throughout the country [25], much alike its impact on the global counterparts [26]. Constant support for mental and psychosocial well-being in different groups during the outbreak should be of highest priority. [9,16]

Demographic variables showcase that males had lesser psychological impact of COVID-19 outbreak as compared to their female counterpart. The impact on females was found to be statistically significant. These findings were similar in the Chinese community where females suffered a greater psychological impact of due to the coronavirus outbreak. [10,27] This also corresponds to previously available extensive epidemiological literature which shows that women are at a higher risk. [28] In our survey, physical co-morbidities were a predictor for higher psychological impact in response to the outbreak, similar to the finds in existing research. [29] An unexpected finding was the non-statistically significance of impact of being a health care worker on psychological impact. This is contrary to existing literature [30] about them being more prone to unfavourable mental health outcomes. This could have been due to low sample size of healthcare professionals representation in the study; thus limiting generalizability of the findings.

However, there are some more limitations to be considered while analysing the study results. First is the inherent design of the study like sampling technique being only restricted to people with internet access and having understanding of English; could also limit generalizability of the study. Second are the concerns of social desirability while responding to questions on mental health issues. Thirdly the study was conducted during a period of lockdown, which can have its own psychological impact and this confounder could not be addressed through the questionnaire used in the study. These issues could have caused under or over reporting in the rate of psychological impact found in the study. Since approximately 20% of the study participants had history of some liver disease, there could be a sampling bias in the study. Moreover, the questionnaire used has not been validated in Indian population earlier. But we felt the timely need of conducting this survey in order to enhance the understanding of psychological concerns and hence a separate validation was not attempted before the study.

Despite the limitations, this study provides the first cross-sectional data on actual level of psychological impact among Indian community; and how mental health of people is affected during a pandemic of this nature. Online surveys (or self-administered questionnaires) have been found as an effective way of assessing problems related to mental health [31,32] and this becomes a prudent method of conducting research in the period of lockdown. Since these findings pertain to the initial period of pandemic in India, a larger longitudinal study should be conducted in the current time to guide policy makers in understanding the psychological impact.

## 5 Conclusion

COVID-19 pandemic has caused a lot of uncertainty in the lives of Indian public, just like their global counterparts. Our survey is one of the first mental health related data from India, during the initial phase of COVID-19 pandemic and indicated that a significant proportion of them have had a psychological impact during the crisis. The factors that predicted higher impact were younger age, being female and having a known physical comorbidity. There is a need for considering mental health issues by the policy makers; while planning interventions to fight the pandemic.
